# Factors Associated with Attrition and Performance Throughout Surgical Training: A Systematic Review and Meta-Analysis

**DOI:** 10.1007/s00268-020-05844-0

**Published:** 2020-10-26

**Authors:** Carla Hope, John-Joe Reilly, Gareth Griffiths, Jon Lund, David Humes

**Affiliations:** 1 Division of Medical Sciences and Graduate Entry Medicine, University of Nottingham, Derby, DE22 3NE UK; 2 Queen’s Medical Centre, Nottingham University Hospitals NHS Trust, Nottingham, NG7 2UH UK; 3 Ninewells Hospital, Dundee, UK; 4 Division of Epidemiology and Public Health, Nottingham Digestive Diseases Centre, School of Medicine, University of Nottingham, Nottingham, UK; 5 National Institute for Health Research Nottingham Digestive Diseases Biomedical Research Unit, Nottingham University Hospitals NHS Trust, E Floor West Block, QMC Campus, Nottingham, NG7 2UH UK

## Abstract

**Background:**

Attrition within surgical training is a challenge. In the USA, attrition rates are as high as 20–26%. The factors predicting attrition are not well known. The aim of this systematic review is to identify factors that influence attrition or performance during surgical training.

**Method:**

The review was performed in line with PRISMA guidelines and registered with the Open Science Framework (OSF). Medline, EMBASE, PubMed and the Cochrane Central Register of Controlled Trials were searched for articles. Risk of bias was assessed using the Newcastle–Ottawa scale. Pooled estimates were calculated using random effects meta-analyses in STATA version 15 (Stata Corp Ltd). A sensitivity analysis was performed including only multi-institutional studies.

**Results:**

The searches identified 3486 articles, of which 31 were included, comprising 17,407 residents. Fifteen studies were based on multi-institutional data and 16 on single-institutional data. Twenty-nine of the studies are based on US residents. The pooled estimate for overall attrition was 17% (95% CI 14–20%). Women had a significantly higher pooled attrition than men (24% vs 16%, *p* < 0.001). Some studies reported Hispanic residents had a higher attrition rate than non-Hispanic residents. There was no increased risk of attrition with age, marital or parental status. Factors reported to affect performance were non-white ethnicity and faculty assessment of clinical performance. Childrearing was not associated with performance.

**Conclusion:**

Female gender is associated with higher attrition in general surgical residency. Longitudinal studies of contemporary surgical cohorts are needed to investigate the complex multi-factorial reasons for failing to complete surgical residency.

**Electronic supplementary material:**

The online version of this article (10.1007/s00268-020-05844-0) contains supplementary material, which is available to authorised users.

## Introduction

Attrition within surgical training is a challenge, in the USA, attrition rates are as high as 20–26% [1, 2]. It is a priority to retain surgical residents to meet the increasing healthcare demand and to reduce the significant costs associated with attrition.

Discrimination in the workplace is protected by US law [3]. Age, sex, disability, race, religion, gender reassignment, sexual orientation, pregnancy and maternity and marriage and civil partnerships are termed ‘protected characteristics’ and relate to personal characteristics or attributes. Differential attainment refers to the differences in performance between groups with and without protected characteristics [4]. The impact of protected characteristics on attrition and performance in general surgery residency is poorly understood.

The impact of gender on attrition from general surgery residency remains unclear. Two meta-analyses reported conflicting findings regarding differences in attrition between male and female general surgery residents [5, 6]. The focus of these meta-analyses was on attrition prevalence and timing as opposed to the impact of protected characteristics. The need for further studies to clarify the role these characteristics play in attrition and performance in general surgical training was highlighted in the 2019 American College of Surgeons (ACS) statement on Harassment, Bullying and Discrimination [7]. Similarly the UK regulatory body, the General Medical Council (GMC), is working to identify areas of inequality to ensure all doctors are treated fairly regardless of protected characteristics [8].

In recent years, there has been a push to increase the diversity of medical students [9]. Consequently, there is a change in the upcoming surgeons of the future, with women now representing over a third of US surgeons in training [10]. Most studies focus on dated cohorts and do not reflect the change in the demographics of present surgical residents.

In order to reduce attrition and ensure that all trainees are facilitated to meet their maximum potential and maintain a successful surgical career, factors affecting failure to complete training or those that adversely affect performance need to be identified.

## Objectives

The aim is to identify factors that influence progression through or completion of surgical training and will address the following:

1. Are there any factors that predict attrition within postgraduate surgical training?
2. Are there any factors that predict performance during postgraduate surgical training?

## Methods

### Protocol registration

This systematic review was performed in line with the Preferred Reporting Items for Systematic Reviews and Meta-Analyses (PRIMSA) [11]. The protocol is available on the Open Science Framework (OFS) at https://osf.io/p5cby.

### Eligibility criteria

The review sought to identify papers evaluating factors that affect attrition or progression through surgical training or identify factors affecting performance within surgical training. We included all types of study published as full papers with no restrictions on the language of or date of publication.

### Exclusion criteria

1. Studies not investigating specialty surgical trainees, e.g. consultants/faculty, non-medical staff, undergraduate training.
2. Studies focused on selection into training.

### Information sources, search and study selection

MEDLINE Ovid, Embase Ovid, PubMed and the Cochrane Central Register of Controlled Trials (CENTRAL) were searched electronically using a mixture of keywords and MeSH terms. The subject strategies for databases were modelled on the search strategy designed for MEDLINE Ovid (Supplemental Fig. 1). We searched the reference lists of included studies for further eligible studies.

Two review authors (CH and JJR) independently and in duplicate performed the title and abstract screening. The full text of all eligible and potentially eligible studies were further evaluated to identify studies meeting the inclusion criteria. Any disagreement was resolved by discussion or where necessary a third reviewer opinion.

### Data collection process

Two review authors (CH and JJR) independently extracted the data. If clarification was needed for any aspect of the included studies, the authors were contacted by email. The primary outcome measures were attrition and performance through training. Attrition was defined as voluntarily or involuntary discontinuation of surgical residency. Protected characteristics (age, sex, ethnicity, and marital and parental status), other factors (personal, workplace/programme, educational/academic) and factors related to performance (examination performance, personality/learning style, operative volume) were extracted from each study. Publication year, country of origin, study size and population, methodology and data source were recorded.

### Risk of bias in individual studies

Methodology checklists for both cohort and case–control studies were reviewed and used to critically appraise and grade the evidence of included studies. Quality was assessed using the Newcastle–Ottawa scale [12].

### Synthesis of results

The results were divided into studies that investigated factors affecting attrition and studies that focused on factors that affected performance. A random effects meta-analysis was performed to generate a pooled estimate of attrition prevalence. Two sensitivity analyses were performed including only multi-institutional studies and studies published after 2008. Between studies, heterogeneity was measured with the I^2^ statistic. I^2^ of greater than 75% was taken as a high level of heterogeneity. Random effects meta-analyses was conducted for sex. In the event of more than one study including the same population of residents, the study with the largest sample was included in the meta-analysis. Subgroup differences were tested using the *z* test. It was not possible to perform a meta-analysis on any other factors due to variation in outcome measurement and study design. It was also not possible to look at attrition worldwide due to the lack of non-US studies. All analyses were performed in Stata version 15 (Stata Corp LP), with a *p* < 0.05 significance level.

## Results

### Study selection

**Fig. 1:**
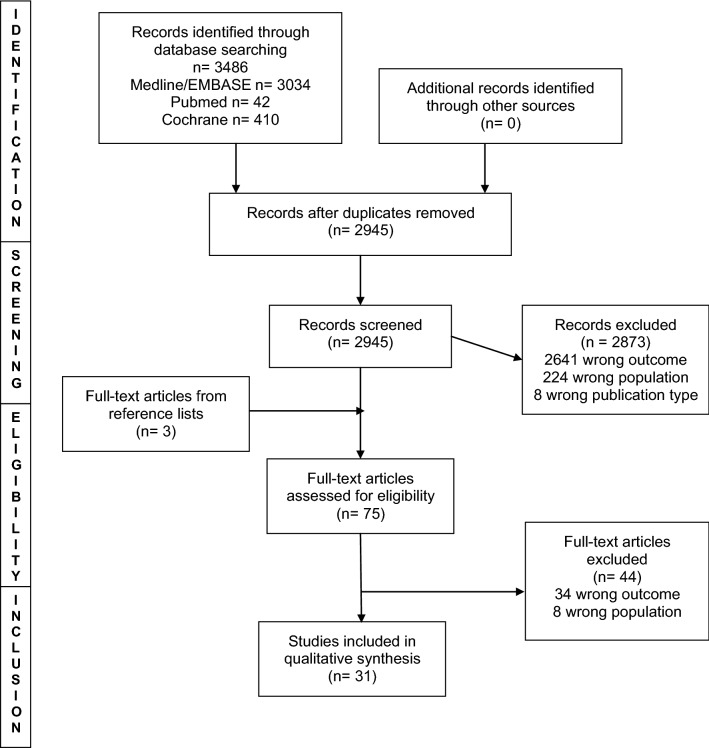
PRISMA flow diagram

**Fig. 2:**
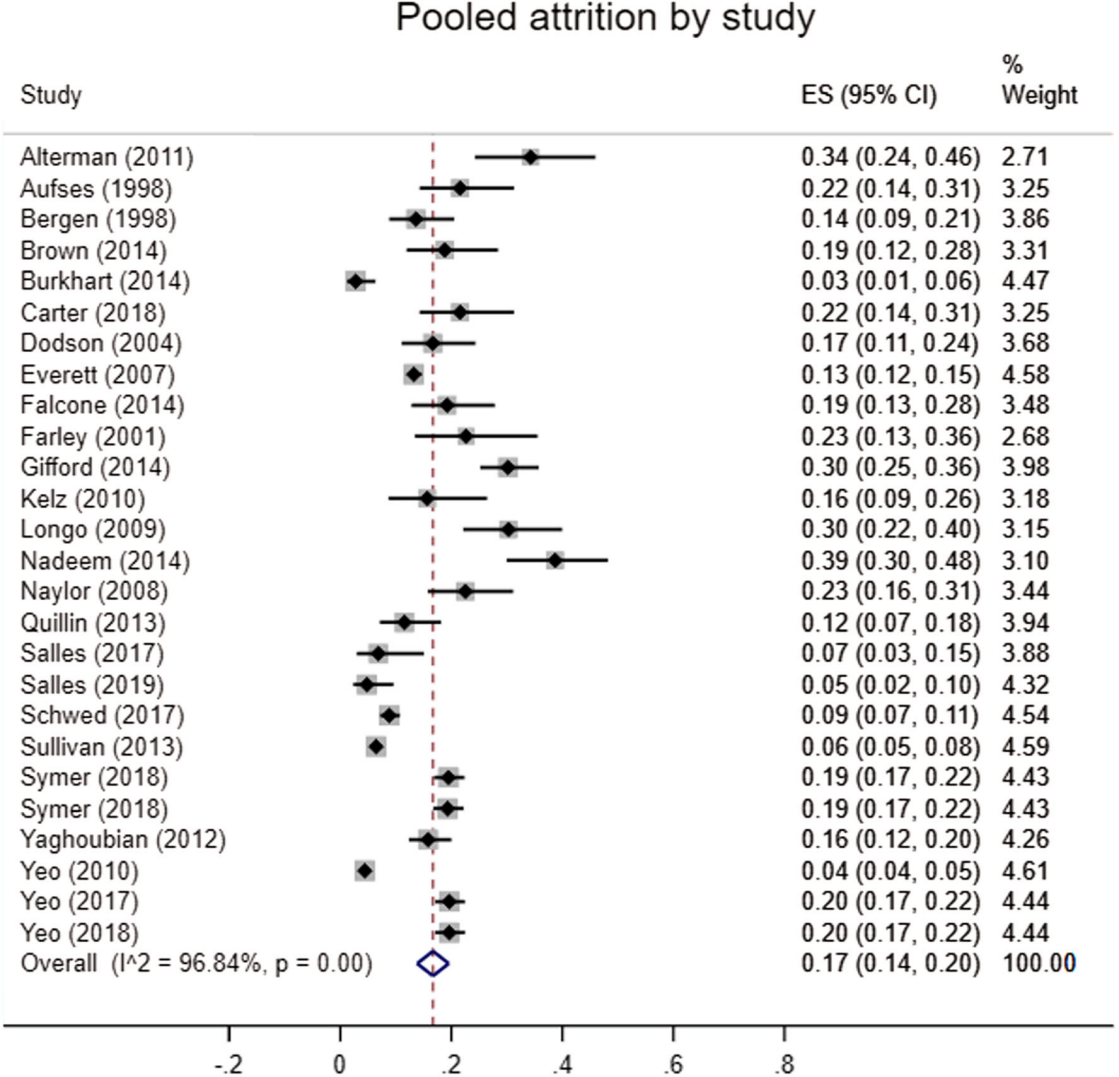
Pooled attrition by study

The searches identified 3486 articles (Fig. 1). The main reason for exclusion on title and abstract screening was wrong outcome or wrong population. Thirty-one studies met the inclusion criteria (Table 1). Twenty-nine of the studies were from the USA, one from Pakistan and one from the UK. In regard to study quality, five studies were at high risk of bias, fifteen moderate risk and eleven low risk (Supplemental Table 1). Twenty-six studies reported attrition prevalence and were included in the meta-analysis, comprising 17,407 residents. The pooled estimate of overall attrition was 17% (95% CI 14–20%) with significant heterogeneity (I^2^ = 96.84%, *p* < 0.001) (Fig. 2). The pooled estimate of attrition was 14% (95% CI 10–17%) on sensitivity analysis of only multi-institutional studies with greater heterogeneity (I^2^ = 98.10%, *p* = 0.00), and therefore, initial analyses are presented (Supplemental Fig. 2). After only including studies published after 2008, the overall attrition remained 17% (95% CI 13–20%, I^2^ = 97.06%, *p* = 0.00) (Supplemental Fig. 3).

**Table 1:** Summary of all included studies

Author	Title	Year	Country	Study size	Population	Methodology	Source	Attrition rate reported
Alterman	The predictive value of general surgery application data for future resident performance	2011	USA	101	General surgery residents 1990–2008 Single institution	Retrospective review	Resident files ABSITE ACGME	Yes
Aufses	The nature and fate of categorical surgical residents who 'drop out'	1998	USA	88	General surgery residents 1982–1995 Single institution	Retrospective review	Resident files Medical school data	Yes
Bergen	Gender-related attrition in a general surgery training program	1998	USA	132	General surgery residents 1984–1996 Single institution	Retrospective review	National Residency Matching Program	Yes
Brown	Pregnancy-related attrition in general surgery	2014	USA	85	General surgery residents 1999–2009 Single institution	Retrospective review	Resident files	Yes
Burkhart	Grit: A marker of residents at risk for attrition?	2014	USA	180	General surgery residents 2012–2013 Multi-institutional	Survey	Grit survey Resident files	Yes
Carter	Women in surgery: A longer term follow-up	2018	USA	108	General surgery residents 1996–2009 Single institution	Retrospective review	Resident files	Yes
Dodson	Why do residents leave general surgery? The hidden problem in today's programs	2004	USA	120	General surgery residents 1990–2003 Single institution	Retrospective review	Resident files	Yes
Everett	General surgery resident attrition and the 80 h workweek	2007	USA	2555	General surgery residents 2001–2004 Multi-institutional	Survey to directors of general surgery residency programme	Survey	Yes
Falcone	Home school dropout: a 20 year experience of the matriculation of categorical general surgery residents	2014	USA	104	General surgery residents 1992–2011 Single institution	Retrospective review	Resident files	Yes
Farley	Whatever happened to the General Surgery graduating class of 2001?	2001	USA	53	General surgery residents 1996–2001 Multi-institutional	Cross-sectional	Resident files	Yes
Gifford	Factors associated with general surgery residents' desire to leave residency programs: A multi-institutional study	2014	USA	288	General surgery residents Multi-institution 2004–2013	Survey across residents	Survey Resident files	Yes
Hayward	Is there gender bias in the evaluation of surgical residents?	1987	USA	144	General surgery residents 1967–1985 Single institution	Retrospective review of faculty evaluations	Resident files	N/A
Kelz	Prevention of Surgical Resident Attrition by a Novel Selection Strategy	2010	USA	64	General surgery residents 2005–2009 Single institution	Retrospective review	Electronic Resident Application System Resident interviews	Yes
Kim	The effect of surgical resident learning style preferences on American board of surgery in-training examination scores	2015	USA	53	General surgery residents 2012–2013 Single institution	Retrospective review	Fleming VARK learning styles inventory ABS in-training examination score	N/A
Leibrant	Has the 80 h work week had an impact on voluntary attrition in general surgery residency programs?	2006	USA	215 programmes	General surgery residents 2003–2004 Multi-institutional	Questionnaire to programme directors	Questionnaire	N/A
Longo	Attrition of categoric general surgery residents: results of a 20 year audit	2009	USA	99	General surgery residents 1986–2006 Single institution	Retrospective review	Resident files	Yes
Nadeem	Attrition in surgical residency programmes: Causes and effects	2014	Pakistan	106	General surgery, orthopaedics, neurosurgery, ENT and urology residents 2005–2011 Single institution	Questionnaire to residents and programme directors	Resident files Survey	Yes
Naylor	Factors Related to Attrition in Surgery Residency Based on Application Data	2008	USA	111	Surgery doesn’t define 1991–2000 Single institution	Retrospective review	Residency application form Annual performance evaluations ABSITE	Yes
Quillin	How residents learn predicts success in surgical residency	2013	USA	130	General surgery residents 1999–2012 Single institution	Retrospective review	ACGME Operative log data ABSQE and ABSCE Resident files	Yes
Salles	Grit as a predictor of risk of attrition in surgical residency	2017	USA	73	General surgery residents 2014–2015 Single institution	Survey to residents	Survey Short grit scale Resident files	Yes
Salles	Social Belonging as a Predictor of Surgical Resident Well-being and Attrition	2019	USA	146	General surgery, cardiothoracic, ENT, vascular, orthopaedics, plastic surgery, urology & neurosurgery residents 2010 2011, 2015 Multi-institutional	Survey to residents	Survey Resident files	Yes
Schwed	Association of general surgery resident remediation and program director attitudes with resident attrition	2017	USA	966	General surgery residents 2010–2015 Multi-institutional	Survey to programme directors	Survey General Surgery Qualifying Examination and General Surgery Qualifying Examination of the ABS	Yes
Scrimgeour	Does the Intercollegiate Membership of the Royal College of Surgeons (MRCS) examination predict ‘on-the-job’ performance during UK higher specialty surgical training?	2018	UK	2750	Higher surgical residents 2007–2016 Multi-institutional	Longitudinal cohort study	Membership of Royal College of Surgeons examination ARCP outcomes	N/A
Sullivan	Surgical residency and attrition: Defining the individual and programmatic factors predictive of trainee losses	2013	USA	2033	General surgery 2008–2009 Multi-institutional	Prospective study	National Study of expectations and Attitudes of Residents in Surgery Survey ABS resident roster	Yes
Symer	The Surgical Personality: Does Surgery Resident Motivation Predict Attrition?	2018	USA	801	General surgery 2007–2008 Multi-institutional	Survey to residents, data review 2016	Behavioural Inhibition/Behaviour Approach scale ABS resident roster	Yes
Symer, Wong	Impact of medical school experience on attrition from general surgery residency	2018	USA	792	General surgery 2007–2008 Multi-institutional	Prospective cohort study	ABS Survey assessing medical school experience	Yes
Wade	Evaluations of surgery resident performance correlate with success in board examinations	1993	USA	48	General surgery 1976–1988 Single institution	Retrospective review	ABSITE Resident files	N/A
Yaghoubian	General surgery resident remediation and attrition: A multi-institutional study	2012	USA	348	General surgery residents 1999–2010 Multi-institution	Retrospective analysis	USMLE ABSITE scores 3rd year medical school surgery performance scores	Yes
Yeo	A national study of attrition in general surgery training: Which residents leave and where do they go?	2010	USA	3959	General surgery residents 2007–2008 Multi-institutional	Retrospective analysis	National Study of Expectations and Attitudes of Residents in Surgery survey	Yes
Yeo	Who Makes It to the End?: A novel predictive model for identifying surgical residents at risk for attrition	2017	USA	836	General surgery residents 2007 Multi-institutional	Prospective cohort study with 8-year follow-up	ABS ABSITE	Yes
Yeo	Association of time to attrition in surgical residency with individual resident and programmatic factors	2018	USA	836	General surgery residents 2007–2008 Multi-institutional	Prospective cohort study With 9-year follow-up data linkage	National Expectations and Attitudes of Residents in Surgery Survey	Yes

*ABS* American Board of Surgery, *ABSITE* American Board of Surgery In-Training examination, *ACGME* Accreditation Council for Graduate Medical Education, *ARCP* Annual Review of Competency Panel, *USMLE* United States Medical Licensing Exam

### Attrition

#### Age

Two out of four studies found no association with age and attrition [1, 13] (Table 2). In the studies that found increasing age to be a risk factor for attrition, age was dichotomised to under and over 29 [14] and under and over 35 years [15] in the analysis. The positive finding in the study by Naylor [14] may be due to the outcome measure which combines attrition with failure to pass the board examination.

**Table 2:** Studies investigating the effect of protected characteristics on attrition (age, sex, race/ethnicity, and marital and parental status)

Age			
Author	Year	Study size	Conclusions
Brown	2014	85	Age not associated with attrition (OR 1.0 95% CI 0.8–1.4)
Yeo	2010	3959	Age not associated with attrition (β 0.05 95% CI -0.03–0.13)
Naylor	2008	111	Age > 29 years associated with attrition (OR 0.11 95% CI 0.02–0.47)
Sullivan	2013	2033	Age ≥ 35 years associated with attrition (OR 0.28 95% CI 0.19–0.39)
*Sex*			
Author	Year	Study size	Conclusions
Alterman	2011	101	No association with gender (not reported)
Aufses	1998	88	No association with gender (32% vs 17%, *p* = 0.12)
Bergen	1998	132	No association with gender (RR 2.26 95% CI 0.96–5.31)
Brown	2014	85	No association with gender (OR 1.0 95% CI 0.2–3.6)
Carter	2018	108	No association with gender (female 22% vs male 19%, *p* = 0.77)
Dodson	2004	120	No association with gender (female 27% vs male 13%)
Falcone	2014	103	No association with gender (female 23.1% vs male 17.9%, *p* = 0.57)
Longo	2009	99	No association with gender (female 39% vs 26% male)
Nadeem	2014	106	No association with gender (female 54.5% vs male 34.5%, *p* = 0.07)
Sullivan	2013	2033	No association with gender (female 7% vs male 6.2%)
Yaghoubian	2012	348	No association with gender (female 47.3% vs male 52.7%, *p* = 0.08)
Yeo	2010	3959	No association with gender (β -0.23 95% CI -0.72–0.30)
Gifford	2014	371	Significant difference between sexes (Female OR 1.9 CI 1.2–3.0)
Symer, Wong	2018	792	Significant difference between sexes (24% of women vs 16% of men left, p 0.01)
Yeo	2017	836	Significant difference between sexes (24% of women vs 17% of men left, *p* = 0.02)
Yeo	2018	836	Significant difference between sexes (Female OR 1.40 95% CI 1.02–1.94)
*Race/Ethnicity*			
Author	Year	Study size	Conclusions
Yeo	2010	3959	No association between race/ethnicity (β -0.09 95% CI -0.59–0.41)
Sullivan	2013	2033	Hispanic residents higher risk of attrition (OR 0.50 95% CI 0.38–0.65) Black residents higher risk of attrition (OR 0.28 95% CI 0.20–0.40)
Symer, Wong	2018	792	Hispanic residents less likely to complete residency (13.1% non-Hispanic vs 7.8% Hispanic completed residency, *p* = 0.04)
Yeo	2017	836	Hispanic residents less likely to complete residency (29% non-Hispanic vs 19% Hispanic completed residency, *p* = 0.03)
Yeo	2018	836	Hispanic residents less likely to complete residency (OR 1.71 95% CI 1.06–2.76)
*Marital and Parental Status*			
Author	Year	Study size	Conclusions
Brown	2014	85	No association with child rearing (OR 1.0 95% CI 0.1–9.6) Association with marital status (Married OR 0.2 95% CI 0.01–0.9)
Sullivan	2013	2033	No association with child rearing (18% with children left vs 15% completed residency, *p* 0.31) No association with marital status (46% married left vs 42% completed residency, *p* 0.34)
Yeo	2010	3959	No association with child rearing (β 0.45 95% CI -0.32–0.98) No association with marital status (OR 0.23 95% CI -0.32–0.78)

#### Gender

**Fig. 3:**
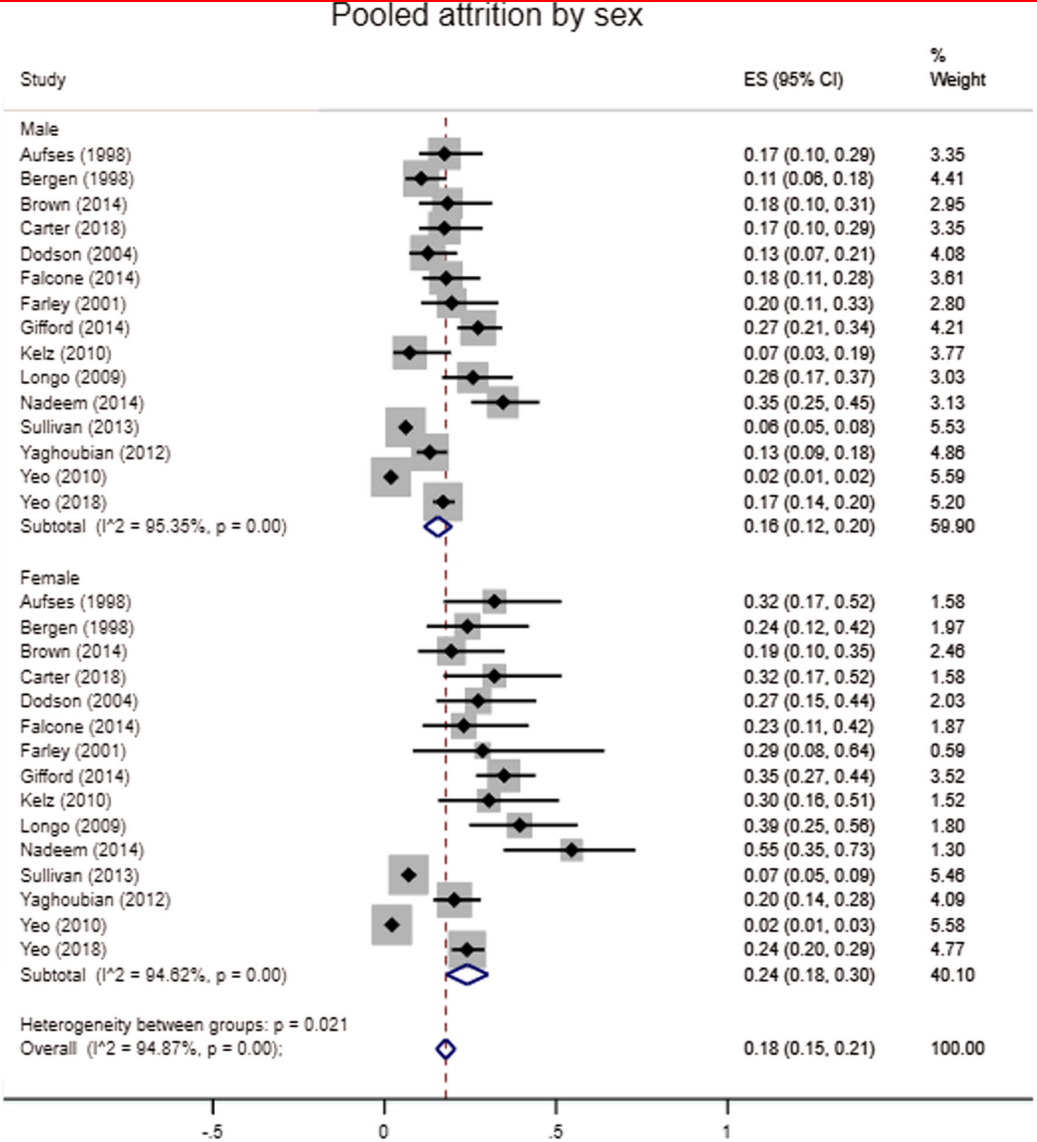
Pooled attrition by sex

The pooled attrition prevalence for male residents on random effect meta-analysis was 16% (95% CI 12–20%), with significant between study heterogeneity (I^2^ = 95.35%, *p* < 0.01) (Fig. 3). The pooled attrition prevalence for women was significantly higher at 24% (95% CI 18–30%, *z* = -4.6832 *p* < 0.001), again with significant heterogeneity (I^2^ = 94.62%, *p* < 0.01). On sensitivity analysis, including only multi-intuitional studies or those published after 2008 did not significantly affect the pooled attrition of male or female residents (Supplemental Fig. 4 and 5).

Four out of 16 studies found a significantly higher attrition amongst female residents [16–19] (Table 2). One reported that women were almost twice as likely to leave training as men (OR 1.9 95% CI 1.2–3.0) [16]. A nine-year follow-up study found there were differences in attrition rate for men and women over time, with similar rates in the first year, but at four years into residency women had significantly higher rates of attrition (21.9% vs 16.3%, *p* = 0.05) [19]. Women also had a higher cumulative attrition (OR 1.40 95% CI 1.02–1.94) [19].

#### Ethnicity/race

Five studies investigated the association between race or ethnicity and attrition [1, 15, 17–19] (Table 2). Four studies reported that Hispanic residents were less likely to complete residency; however, 3 of these studies were based on the same population of residents [15, 17–19]. Therefore, it was not possible to perform a meta-analysis of the data. One study found that while white race was not associated with higher completion rates across both genders (69.7% completion vs 65.8% non-completion, *p* = 0.34) [18], on subgroup analysis of women, white women had lower non-completion rates than non-white women (20% vs 30% *p* = 0.08).

#### Marital and parental status

None of the studies found an association between parental status and attrition [1, 13, 15]; this included two large multi-institutional studies (Table 2). Only one study found those that were married were less likely to leave training, this was a small single-institutional study of 85 residents from 1999 to 2009 [13].

#### Personal factors

There was no association between attrition and ‘grit’ [20, 21] social belonging [22] or motivational personality traits [23] (Table 3). Grit was defined as perseverance and passion for long-term goals. The number of residents that did not complete training in these studies was small and therefore limits the power of statistical analysis. Quillin et al. reported that residents who learn by observation are more likely to leave the programme and opt for a non-surgical specialty [24].

**Table 3:** Studies investigating the effect of other factors on attrition (personal, workplace and programme and educational/academic factors)

Personal factors			
Author	Year	Study size	Conclusions
Burkhart	2014	180	No significant association with grit (*p* = 0.246)
Salles	2017	73	No significant association with grit (β 85.83, *p* = 0.999)
Salles	2015	146	No significant association with lack of belonging (*r* = 0.15, *p* = 0.1846)
Symer	2018	801	No significant association with motivational personality traits (*p* = 0.51)
Quillin	2013	130	Association with learning style (*p* = 0.0467)
*Workplace and programme factors*			
Author	Year	Study size	Conclusions
Everett	2007	2555	Association with 80 h workweek (0.6 lost/programme/year pre vs 0.8 lost/programme/year post, *p* = 0.0414)
Leibrant	2006	215	No association with 80 h work week (0.7 lost/programme/year pre vs 0.8 lost/programme/year post)
Sullivan	2013	2033	Association with early postgraduate year (9.4% PGY-1 left vs 4.5% PGY-2, *p* < 0.001) Association with regional location (Northeast HR 2.39 vs South, *p* = 0.006)
Yeo	2010	3959	Association with early postgraduate year (β -0.82 95% CI -1.06- -0.58)
Symer, Wong	2018	792	Association with larger programme size (25.4% from large programme completed vs 34% small programme, *p* = 0.03)
Yeo	2017	836	Association with larger programme size (24% from large programme left vs 18% small programmes, *p* = 0.03) Association with military programmes vs academic programmes vs community programmes (35% vs 20% vs 17%, *p* = 0.03)
Yeo	2018	836	Association with military programmes vs academic programmes (OR 2.68 95% CI 1.36–5.29)
Yaghoubian	2012	348	No association with remediation (20% vs 15%, *p* = 40)
*Educational and academic factors*			
Author	Year	Study size	Conclusions
Farley	2001	53	No association with applicant ranking (*p* = 0.18)
Falcone	2014	103	No association with place of medical degree (*p* = 0.89)
Yaghoubian	2012	348	No association with place of medical degree (0% vs 5.3% foreign medical graduate,*p* = 0.09) Association with PGY-3 ABSITE score (*p* = 0.04)
Alterman	2011	101	Association with special skill on medical school application (OR 3.59 95% CI 1.035–11.95) and medical school grade point average (*p* = 0.023) Association with residency interview score (OR 188.27 95% CI 3.757–9435.405), STEP1 score (*p* = 0.001) and ABSITE score (*p* < 0.001)
Naylor	2008	111	Association with residency application—comments in the dean’s letter (OR 4.57 95% CI 2.00–10.43), participation in team sports (OR 4.96 95% CI 1.36–18.05) Association with merit scholarship (OR 0.25 95% CI 0.08–0.78)
Symer, Wong	2018	792	Association with experience in surgical clerkship—perception that medical school faculty were happy with their careers (OR 0.57 95% CI 0.34–0.96), those that got along well with attending surgeons during medical school (OR 2.93 95% CI 1.34–6.39)

#### Workplace and programme factors

Eight studies reported the impact of work place factors on failure to complete general surgery residency [1, 15, 17–19, 25–27] (Table 3). Early postgraduate year [1, 15], larger programme size [17, 18] and military programmes [18, 19] were found to be associated with higher attrition.

#### Educational and academic factors

Six studies investigated medical school factors affecting completion of residency [14, 17, 25, 28–30] (Table 3). Two studies reported an association between ABSITE score and attrition [25, 30]. Residents who felt medical school faculty were happy with their surgical careers were less likely to experience attrition [17], while those who got along well with attending surgeons during medical school had higher odds of attrition. Protective factors on the residency application were comments in the dean’s letter, participation in team sports [14] and residency interview score [30].

#### Performance

Six studies focused on factors that predicted performance throughout surgical residency [20, 24, 31–34] (Table 4). Performance included examination scores, operative case volume and in-training evaluations. Childrearing was not associated with operative case volume or examination performance [13]. Factors reported to affect US postgraduate surgical examination performance were learning preference [31] and faculty evaluation of clinical performance [34]. However, these studies are based on small sample sizes. The only UK-based study investigated whether postgraduate examination scores are a predictor of performance throughout UK surgical training [32]. Non-white ethnicity and examination performance were found to be independent predictors of unsatisfactory performance.

**Table 4:** Studies looking at factors that predict performance

Author	Year	Study size	Conclusions
Brown	2014	85	No association with childrearing and operative case volume (men *p* = 0.40, women *p* = −93) or board pass rates (men *p* = 0.76, women *p* = 0.50)
Burkhart	2014	180	No association between grit score and ABSITE performance (*p* = 0.891)
Hayward	1987	144	No association with gender or place of medical degree (not reported)
Quillin	2013	130	No association with learning style and first time pass rate on the ABSQE (*p* = 0.615) or the ABSCE (*p* = 0.510) Association with learning style and operative cases volume (*p* = 0.0467)
Kim	2015	53	Association with learning preferences and ABSITE performance (*p* = 0.03)
Scrimgeour	2018	2750	Association with non-white ethnicity (OR 1.36 95% CI 1.08–1.71), MRCS pass score (OR 0.98 95% CI 0.98–1.00) and MRCS attempt number (OR 1.50 95% CI 1.16–1.94) with unsatisfactory ARCP outcome
Wade	1993	48	Association with clinical performances and first time pass rate of ABS (*p* < 0.005) Association with ABSITE performance and first time pass rate of the certifying examination (*p* < 0.001)

## Discussion

This is the first study to report the association between protected characteristics and attrition and performance during surgical training. Overall, of the studies included in our systematic review 25 reported factors associated with progression or completion of surgical training and seven focused on factors affecting performance. The pooled attrition rate was high at 17% which causes a burden to residency programmes and existing residents. Efforts should be made to retain residents and to reduce the financial and training implications of attrition. Worryingly given the changing demographic of surgical trainees, rates of attrition were higher in women.

The limitations of this study are related to the included studies, the majority of which are conducted in a single institution which increases bias and reduces generalisability. A significant finding that limits generalisability to current surgical trainees is that fourteen of the studies include cohorts that started training over 20 years ago. During this time, training requirements and assessment processes have changed, as has the population of surgical trainees with an increase in female trainees. However, on sensitivity analysis including only studies published since 2008 did not affect the overall pooled attrition or that of attrition by gender. Nine of the included studies rely on survey data which are subject to response and recall bias. Also, as all but two of the included studies are from the USA, attrition rates and factors affecting this in other countries have not been investigated.

Attrition rates in general surgery residency remain higher than other surgical specialities [35–37]. In a study of Canadian surgical residents, 26.8% were considering leaving their training programme with poor work–life balance cited as the main reason [38]. This study provides clarity regarding the impact of resident gender on attrition after two previous meta-analyses reported differing findings [5, 6]. We found a significantly higher attrition rate for female residents on pooled meta-analysis. This is consistent with a meta-analysis that found female residents had a 25% pooled attrition rate compared to 15% of men [6]. This finding is not unique to general surgery; higher attrition rates for female residents have also been reported in neurosurgery [39, 40] and orthopaedics [36].

The findings regarding the impact of Hispanic ethnicity and attrition require further investigation. As three of the four studies reporting higher attrition amongst Hispanic residents are based on the same population, it is not possible to make firm conclusions. However, higher attrition for Hispanic residents has been reported across other specialities. A 2019 study of US emergency medicine residents found that a significantly greater proportion of Hispanic residents left the programme compared to white residents [41]. They also reported a higher rate of dismissal for Hispanic residents compared to Asian and white residents. The fact that Hispanic residents are an underrepresented group in postgraduate medicine may result in less access to role models they can identify with which may impede residency satisfaction [42]. Residents of non-white ethnicity were less likely to feel they fit in their residency programme which may partly explain the higher attrition [43].

Half of the studies found that age is associated with increased attrition; in both of these studies, age was dichotomised to an arbitrary number which may influence the findings [14, 15]. All three of the studies that analysed the effect of parental status find no increased rates [1, 13, 15]. These findings are reassuring given the increasing number of female surgical trainees and increasing acceptance of childrearing during residency. One study found that while the perception of negative attitudes towards pregnancy during training has decreased over time, some stigma persists [44]. It additionally reported that those who had graduated from medical school more recently were more likely to have a pregnancy during training than their older counterparts. The finding that childrearing does not affect attrition or performance should encourage residency programmes to develop clear guidance regarding parental leave, as in a recent study only 3.8% of residents were able to correctly identify the American Board of Surgery policy and felt unsupported [45].

The studies that focused on performance vary greatly in design and outcome measure. As with attrition, there was no association between childrearing or ‘grit’ and performance. The definition of performance is not uniform across studies and this limits interpretation. Four of these studies are based on populations commencing surgical residency more than 20 years ago, in one case from 1967. A 2017 study outlines the different assessment tools used during residency and highlights the lack of effective tools to measure competence [46]. Further studies investigating the relationship between attrition and performance using standardised measures of performance are warranted.

## Conclusion

Female residents have higher attrition than male residents in general surgery. Marital and parental status are not associated with increased risk of attrition in general surgery residency. Longitudinal studies of contemporary surgical cohorts are needed to investigate the complex and multi-factorial reasons for failing to complete surgical residency internationally.

## Electronic supplementary material

Below is the link to the electronic supplementary material.

Supplementary file1 (DOCX 13 kb)

Supplementary file2 (DOCX 16 kb)

Supplementary file3 (TIF 1388 kb)

Supplementary file4 (TIF 832 kb)

Supplementary file5 (TIF 859 kb)

Supplementary file6 (TIF 1117 kb)
