# Supplementary material for: Factors Associated with Attrition and Performance Throughout Surgical Training: A Systematic Review and Meta-Analysis
Source: World J Surg. 2020 Oct 26;45(2):429–42. doi: 10.1007/s00268-020-05844-0 (PMC7773620; doi:10.1007/s00268-020-05844-0)
Supplement: Supplementary file 1 — Supplementary file1 (DOCX 13 kb) [file 268_2020_5844_MOESM1_ESM.docx]

Supplemental Figure 1: Search strategy for Medline

| 38 | (surgeon or "surgical trainee" or "surgical training" or "surgical residency").mp. [mp=ti, ab, hw, tn, ot, dm, mf, dv, kw, fx, dq, nm, kf, ox, px, rx, ui, sy] |
| --- | --- |
| 39 | (surgical registrar or "specialty trainee" or "core trainee").mp. [mp=ti, ab, hw, tn, ot, dm, mf, dv, kw, fx, dq, nm, kf, ox, px, rx, ui, sy] |
| 40 | exp "Internship and Residency"/ or exp Clinical Competence/ |
| 41 | "specialty training".mp. or exp Specialization/ or exp Education, Medical, Graduate/ or exp Clinical Competence/ |
| 42 | exp Educational Measurement/st [Standards] |
| 43 | underperform*.mp. |
| 44 | (differential attainment or "attainment gap").mp. [mp=ti, ab, hw, tn, ot, dm, mf, dv, kw, fx, dq, nm, kf, ox, px, rx, ui, sy] |
| 45 | (extend* or additional training).mp. [mp=ti, ab, hw, tn, ot, dm, mf, dv, kw, fx, dq, nm, kf, ox, px, rx, ui, sy] |
| 46 | (difficulty or struggl*).mp. [mp=ti, ab, hw, tn, ot, dm, mf, dv, kw, fx, dq, nm, kf, ox, px, rx, ui, sy] |
| 47 | exp Bias/ or bias.mp. |
| 48 | (ethnicity or race).mp. [mp=ti, ab, hw, tn, ot, dm, mf, dv, kw, fx, dq, nm, kf, ox, px, rx, ui, sy] |
| 49 | (ethnicity or race).mp. [mp=ti, ab, hw, tn, ot, dm, mf, dv, kw, fx, dq, nm, kf, ox, px, rx, ui, sy] |
| 50 | prejudice.mp. or exp Prejudice/ |
| 51 | (matriculation or completion).mp. [mp=ti, ab, hw, tn, ot, dm, mf, dv, kw, fx, dq, nm, kf, ox, px, rx, ui, sy] |
| 52 | attrition.mp. |
| 53 | ("annual review of competency progression" or "arcp" or "rita" or "record of in training assessment").mp. [mp=ti, ab, hw, tn, ot, dm, mf, dv, kw, fx, dq, nm, kf, ox, px, rx, ui, sy] |
| 54 | 38 or 39 |
| 55 | 40 or 41 or 42 |
| 56 | 43 or 44 or 45 or 46 or 47 or 50 or 51 or 52 or 53 |
| 57 | 54 and 55 and 56 |
| 58 | remove duplicates from 57 |
