# Supplementary material for: Factors Associated with Attrition and Performance Throughout Surgical Training: A Systematic Review and Meta-Analysis
Source: World J Surg. 2020 Oct 26;45(2):429–42. doi: 10.1007/s00268-020-05844-0 (PMC7773620; doi:10.1007/s00268-020-05844-0)
Supplement: Supplementary file 2 — Supplementary file2 (DOCX 16 kb) [file 268_2020_5844_MOESM2_ESM.docx]

Supplemental Table 1. Quality assessment of cohort studies using Newcastle-Ottawa Scale.

| Study | Representativeness of the exposed cohort | Selection of the non-exposed cohort | Ascertainment of exposure | Demonstration that outcome of interest was not present at start of study | Comparability of cohorts on the basis of the design or analysis | Assessment of outcome | Was follow-up long enough for outcomes to occur | Adequacy of follow up of cohorts | Total score |
| --- | --- | --- | --- | --- | --- | --- | --- | --- | --- |
| Alterman | C | A | A | A | C | B | A | A | 6 |
| Aufses | C | A | A | A | C | B | A | A | 6 |
| Bergen | C | A | A | A | C | B | A | B | 6 |
| Brown | C | A | A | A | A | B | A | A | 7 |
| Burkhart | C | A | C | A | B | B | B | C | 4 |
| Carter | C | A | A | A | B | B | A | A | 7 |
| Falcone | C | A | A | A | C | B | A | A | 6 |
| Hayward | C | A | A | A | B | B | A | B | 6 |
| Kelz | C | A | A | A | A | B | A | A | 7 |
| Kim | C | A | B | A | B | B | B | B | 6 |
| Longo | C | A | A | A | C | B | A | A | 6 |
| Naylor | C | A | A | A | A | B | A | B | 7 |
| Quillin | C | A | A | A | A | B | A | A | 7 |
| Salles (2017) | C | A | C | A | B | D | B | C | 3 |
| Salles (2019) | C | A | C | A | B | D | B | B | 4 |
| Schwed | A | A | A | A | A | B | B | B | 7 |
| Scrimgeour | A | A | A | A | A | B | A | A | 8 |
| Sullivan | A | A | A | A | C | B | A | B | 7 |
| Symer | A | A | A | A | A | B | A | A | 8 |
| Symer, Wong | A | A | B | A | A | B | A | A | 8 |
| Wade | C | A | A | A | B | B | A | A | 7 |
| Yaghoubian | C | A | A | A | C | B | A | D | 5 |
| Yeo (2010) | A | A | A | A | A | B | A | B | 8 |
| Yeo (2017) | A | A | A | A | A | B | A | A | 8 |
| Yeo (2018) | A | A | A | A | A | B | A | A | 8 |

Supplemental table 1.1 Quality assessment of Cross Sectional Studies using Newcastle-Ottawa Scale

| Study | Representativeness of the trainee population | Ascertainment of exposure | Comparability of cohorts on the basis of the design or analysis | Assessment of outcome | Adequacy of response rate | Total score |
| --- | --- | --- | --- | --- | --- | --- |
| Dodson | C | A | C | B | A | 3 |
| Everett | A | A | C | B | C | 3 |
| Farley | C | A | C | B | C | 2 |
| Gifford | C | A | A | B | C | 3 |
| Leibrant | A | A | C | B | B | 4 |
| Nadeem | C | A | C | B | B | 3 |
