# Supplementary figures and images for: Factors Associated with Attrition and Performance Throughout Surgical Training: A Systematic Review and Meta-Analysis
Source: World J Surg. 2020 Oct 26;45(2):429–42. doi: 10.1007/s00268-020-05844-0 (PMC7773620; doi:10.1007/s00268-020-05844-0)

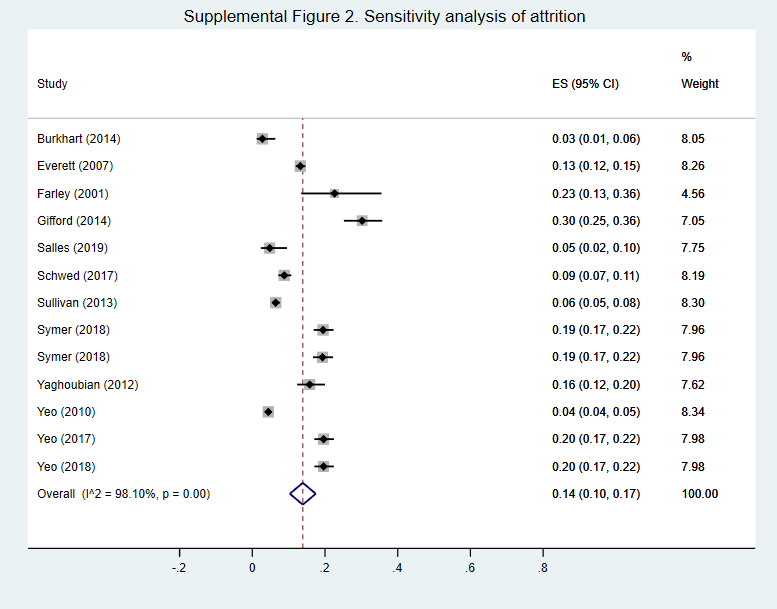

Supplement: Supplementary file 3 — Supplementary file3 (TIF 1388 kb) [file 268_2020_5844_MOESM3_ESM.tif]

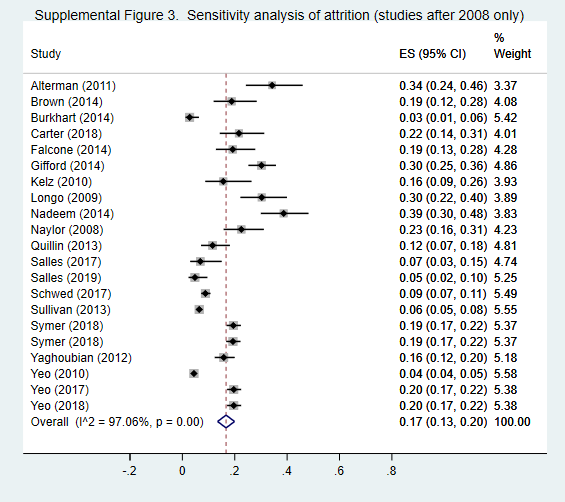

Supplement: Supplementary file 4 — Supplementary file4 (TIF 832 kb) [file 268_2020_5844_MOESM4_ESM.tif]

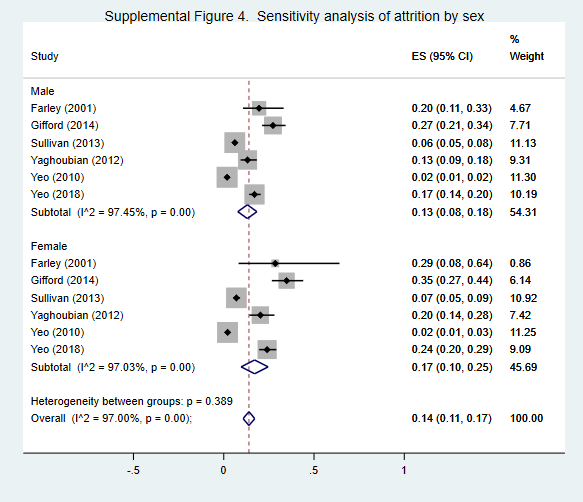

Supplement: Supplementary file 5 — Supplementary file5 (TIF 859 kb) [file 268_2020_5844_MOESM5_ESM.tif]

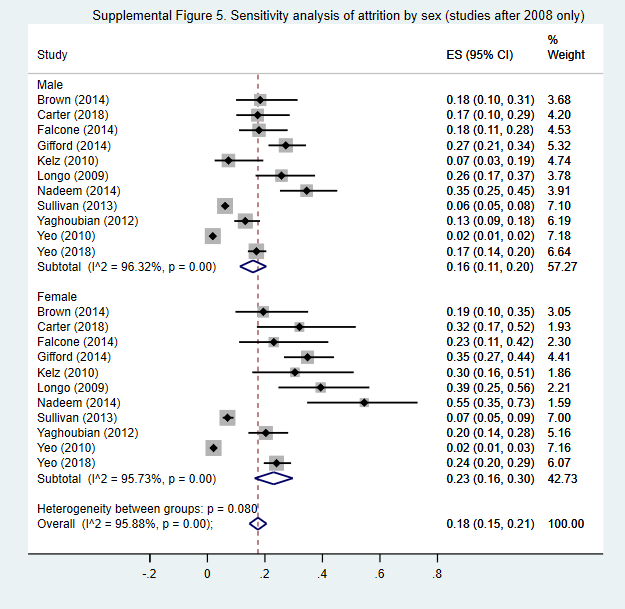

Supplement: Supplementary file 6 — Supplementary file6 (TIF 1117 kb) [file 268_2020_5844_MOESM6_ESM.tif]
